# Granule Leakage Induces Cell-Intrinsic, Granzyme B-Mediated Apoptosis in Mast Cells

**DOI:** 10.3389/fcell.2021.630166

**Published:** 2021-11-08

**Authors:** Sabrina Sofia Burgener, Melanie Brügger, Nathan Georges François Leborgne, Sophia Sollberger, Paola Basilico, Thomas Kaufmann, Phillip Ian Bird, Charaf Benarafa

**Affiliations:** ^1^Institute of Virology and Immunology (IVI), Mittelhäusern, Switzerland; ^2^Department of Infectious Diseases and Pathobiology, Vetsuisse Faculty, University of Bern, Bern, Switzerland; ^3^Graduate School for Cellular and Biomedical Science, University of Bern, Bern, Switzerland; ^4^Theodor Kocher Institute, Department of Preclinical Medicine, Faculty of Medicine, University of Bern, Bern, Switzerland; ^5^Institute of Pharmacology, Department of Preclinical Medicine, Faculty of Medicine, University of Bern, Bern, Switzerland; ^6^Department of Biochemistry and Molecular Biology, Monash Biomedicine Discovery Institute, Monash University, Clayton, VIC, Australia

**Keywords:** serpins, lysosomal peptidases, cell death, granzyme B, lysosomal permeabilization, mast cells, serine protease

## Abstract

Mast cells are multifunctional immune cells scattered in tissues near blood vessels and mucosal surfaces where they mediate important reactions against parasites and contribute to the pathogenesis of allergic reactions. Serine proteases released from secretory granules upon mast cell activation contribute to these functions by modulating cytokine activity, platelet activation and proteolytic neutralization of toxins. The forced release of granule proteases into the cytosol of mast cells to induce cell suicide has recently been proposed as a therapeutic approach to reduce mast cell numbers in allergic diseases, but the molecular pathways involved in granule-mediated mast cell suicide are incompletely defined. To identify intrinsic granule proteases that can cause mast cell death, we used mice deficient in cytosolic serine protease inhibitors and their respective target proteases. We found that deficiency in Serpinb1a, Serpinb6a, and Serpinb9a or in their target proteases did not alter the kinetics of apoptosis induced by growth factor deprivation *in vitro* or the number of peritoneal mast cells *in vivo*. The serine protease cathepsin G induced marginal cell death upon mast cell granule permeabilization only when its inhibitors Serpinb1a or Serpinb6a were deleted. In contrast, the serine protease granzyme B was essential for driving apoptosis in mast cells. On granule permeabilization, granzyme B was required for caspase-3 processing and cell death. Moreover, cytosolic granzyme B inhibitor Serpinb9a prevented caspase-3 processing and mast cell death in a granzyme B-dependent manner. Together, our findings demonstrate that cytosolic serpins provide an inhibitory shield preventing granule protease-induced mast cell apoptosis, and that the granzyme B-Serpinb9a-caspase-3 axis is critical in mast cell survival and could be targeted in the context of allergic diseases.

## Introduction

Developing from bone marrow precursors, mature mast cells are scattered in tissues and found strategically placed in the proximity of blood vessels, nerves, hair follicles and mucosal surfaces. Since their discovery by Paul Ehrlich 140 years ago, physiological and pathological functions of mast cells have been debated (Maurer et al., 2003; Bradding and Pejler, 2018). The textbook view is that mast cells contribute prominently to IgE-mediated allergic reactions and control parasite infections via the rapid release of mast cell granule contents (Galli et al., 2008). Beyond essential contributions to allergy and the control of helminth infection, novel functions linked to degranulation of mast cells have been demonstrated in innate and adaptive immune responses against various microbes, neoplasia, and chronic non-allergic inflammatory diseases (Galli et al., 2015; Oskeritzian, 2015).

Among the preformed factors released by degranulation of mast cells, specialized proteases act to amplify or dampen inflammation and, importantly, to inactivate endogenous and exogenous toxins (Metz et al., 2006; Piliponsky et al., 2008, 2012; Akahoshi et al., 2011; Galli et al., 2015; Waern et al., 2016). Among these granule proteases are chymases, which are serine proteases that evolved by gene duplication from a common ancestor shared with granzymes, cathepsin G (CatG) and complement factor D. These protease genes are located on three conserved loci in vertebrates (Ahmad et al., 2014). Mast cell granules also store soluble α- and β-tryptases, which are serine proteases that evolved from membrane-bound γ-tryptases independently of chymases (Trivedi et al., 2007). Finally, mast cell granules also contain other serine proteases such as granzyme B (GzmB) and CatG, which are not restricted to mast cells. Thus, mast cells store a range of serine proteases in their granules, and these proteases may also induce proteolysis and cell suicide if released from granules into the cytosol.

Protease inhibitors such as clade B serpins have a nuclear and/or cytoplasmic intracellular localization and promote survival of neutrophils, NK and activated cytotoxic T cells (Kaiserman and Bird, 2010; Benarafa and Simon, 2017). The specific function of intracellular serpins in mast cell homeostasis has not been elucidated but expression analysis from the literature and publicly available resources support the hypothesis that intracellular serpins may provide a survival shield in mast cells against their own granule proteases. Indeed, the cytosolic inhibitor SERPINB6 is expressed in mast cells in the lung, skin and tonsils, as well as in mastocytoma and systemic mastocytosis (Strik et al., 2004). SERPINB6 forms inhibitory complexes with monomeric β-tryptase (Strik et al., 2004) and is one of the best-characterized inhibitors of CatG (Scott et al., 1999). In addition to SERPINB6, mast cells express SERPINB1, a related cytosolic serpin that inhibits CatG and chymase, as well as other leukocyte granule proteases such as neutrophil elastase, proteinase-3, and granzyme H (Cooley et al., 2001; Benarafa et al., 2002; Wang et al., 2013). SERPINB9 inhibits GzmB and is expressed in natural killer cells, cytotoxic T lymphocytes, dendritic cells as well as mast cells (Bird et al., 1998; Hirst et al., 2003; Bladergroen et al., 2005). Here, we used mice deficient in Serpinb1a (*Sb1a*^–/–^), Serpinb6a (*Sb6a*^–/–^), Serpinb9a (*Sb9a*^–/–^), and their target proteases to investigate serpin function in mast cell homeostasis and survival upon granule permeabilization and growth factor deprivation *in vitro*. We identify a cytoprotective function for these serpins and a major function for GzmB in inducing mast cell apoptosis following cell-intrinsic granule leakage.

## Results

### Serine Proteases and Caspases Induce Cell Death in Mouse and Human Mast Cells

**FIGURE 1:**
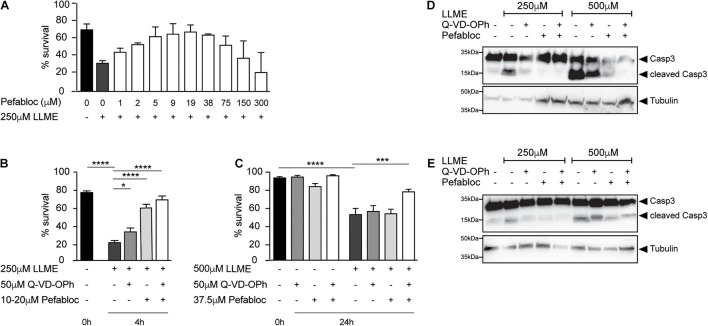
Serine protease- and caspase-dependent cell death in human and mouse mast cells. **(A–C)** Survival of mast cells treated with L-leucyl-L-leucine methyl ester (LLME) in presence of indicated concentrations of serine protease inhibitor (Pefabloc) and pancaspase inhibitor (Q-VD-OPh). Cell viability was determined by flow cytometry as shown in Supplementary Figure 3. Data are shown as mean ± SEM. **(A)** WT mouse BMDMCs (*n* = 3). **(B)** BMDMCs of WT mice (*n* = 10). **(C)** Human mast cell line HMC1.2 (*n* = 7–9). **(B,C)** Data are shown as mean ± SEM and were analyzed by one-way ANOVA (****p < 0.0001; ***p < 0.001; *p < 0.05; ns, not significant). **(D,E)** Western blot analysis of caspase-3 cleavage in WT BMDMCs treated with LLME and inhibitors. **(D)** cell lysates and **(E)** cell lysate and supernatant combined are shown. Fifteen μg of protein was loaded in each lane.

Permeabilization of granules with L-leucyl-L-leucine methyl ester (LLME), a well-characterized lysosomotropic agent, induces cell death in various leukocytes containing granules (Thiele and Lipsky, 1986, 1990a,b). Melo et al. (2011a, 2015) reported that mast cells are sensitive to LLME-induced death, but the precise molecular mechanisms and whether proteases are involved remain unclear. We replicated their findings showing that treatment of mouse bone marrow-derived mast cells (BMDMCs) with LLME rapidly induces cell death (Figure 1A). Inhibition of serine proteases with Pefabloc [a.k.a. 4-benzenesulfonyl fluoride hydrochloride (AEBSF)] inhibited cell death in a dose-dependent manner with optimal concentrations between 10 and 40 μM, although it was toxic at higher concentrations (Figure 1A). Inhibition of caspases with Q-VD-OPh also reduced death of BMDMCs treated with LLME, with or without Pefabloc, suggesting multiple proteases operate in this cell death pathway (Figure 1B). In addition, we observed a synergistic pro-survival effect of the inhibitors of serine proteases and caspases in the human mast cell line HMC-1.2 treated with LLME (Figure 1C). Importantly, LLME induced a strong activation of caspase-3, which was inhibited by both Q-VD-OPh and by Pefabloc (Figures 1D,E), suggesting that granule serine proteases may contribute to caspase-3 activation leading to apoptosis.

### Expression of Proteases and Corresponding Cytosolic Inhibitors in Mouse Bone Marrow-Derived Mast Cells

**FIGURE 2:**
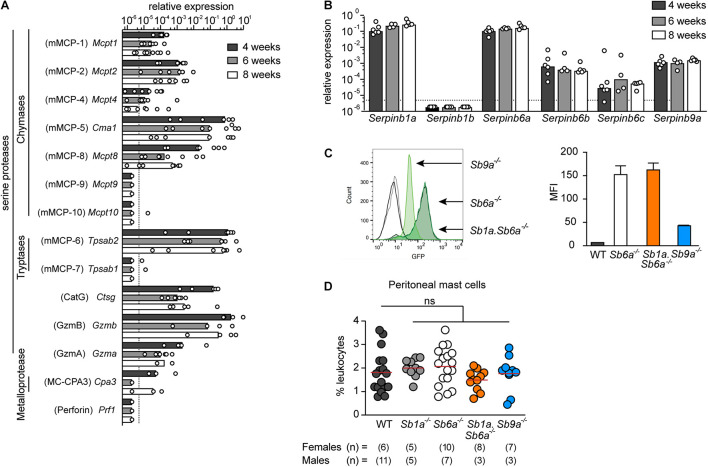
Expression of serine proteases and clade B serpins in BMDMCs. **(A)** Relative mRNA expression of mouse mast cell proteases (mMCP), including chymases (*Mcpt*), tryptases (*Tpsab*), cathepsin G (*Ctsg*), granzymes (*Gzm*), carboxypeptidase 3 (*Cpa3*); and expression of perforin (*Prf1*) in WT mouse BMDMCs at indicated time of *in vitro* differentiation with IL-3. **(B)** Relative mRNA expression of mouse serpin homolog genes in WT mouse BMDMCs at indicated time of *in vitro* differentiation with IL-3. Data are shown as mean relative expression (bars) with individual BMDMC preparation (dots) (*n* = 4–6). Data are shown as median with range. **(C)** Representative flow cytometry histogram (left) and mean ± SD (right) of mean fluorescence intensity (MFI) of reporter GFP signal in BMDMCs of *Sb6a*^–/–^, *Sb1a.Sb6a*^–/–,^ and *Sb9a*^–/–^ mice. **(D)** Percentage of mast cells (IgE^+^, FcεR1a^+^, c-Kit^+^) in peritoneal cavity of WT, *Sb1a*^–/–^, *Sb6a*^–/–^, *Sb1a.Sb6a*^–/–^, and *Sb9a*^–/–^ female and male mice. Data were analyzed by one-way ANOVA and showed no difference relative to WT mice; data are shown as median (red line) and for individual mice (*n* = 5–11/sex/genotype).

To start identifying the protease(s) responsible for inducing cell death, we then evaluated the expression of a broad range of serine proteases in WT BMDMCs during *in vitro* differentiation with recombinant mouse IL-3 for 4, 6, and 8 weeks. mRNA expression levels in WT BMDMCs revealed relatively high expression of the mast cell-restricted serine proteases chymases mMCP-2 and mMCP-5, and the tryptase mMCP-6, whereas mMCP-4, -7, -9, and -10 expression was weak or not detected (Figure 2A). Furthermore, we found that serine proteases cathepsin G (CatG), granzyme B (GzmB), and granzyme A (GzmA), which are not restricted to mast cells, are expressed at levels similar to MCP-2, independently of the maturation state of the BMDMCs. Perforin expression, in contrast, was not detected. Expression of the metalloprotease carboxypeptidease 3 (MC-CPA3) was also detected (Figure 2A).

Since BMDMCs expressed appreciable mRNA levels of CatG, GzmA, and GzmB, we investigated the expression of their respective cytosolic inhibitors in WT BMDMCs during *in vitro* differentiation. We found that two CatG inhibitors *Serpinb1a* (Benarafa et al., 2002) and *Serpinb6a* (Sun et al., 1997) had the highest expression levels. In contrast, transcription of *Serpinb1b*, which also inhibits CatG, was not detectable. Expression of the inhibitors of GzmA and GzmB, *Serpinb6b* (Kaiserman et al., 2014) and *Serpinb9a* (Sun et al., 1997), respectively, were also strongly expressed in all preparations and time points. Expression of *Serpinb6c*, which has no known target protease identified to date, was lower and not consistently detectable (Figure 2B). Previous studies reported that GzmA protein is not expressed in mast cells (Pardo et al., 2007; Ronnberg et al., 2014). Using a GzmA-specific antibody and intracellular staining, we found specific staining of GzmA in wild-type NK cells, but not peritoneal mast cells nor BMDMCs, suggesting that GzmA is post-transcriptionally regulated in mast cells (Supplementary Figures 1A, 2). Because *Sb6a*^–/–^ and *Sb9a*^–/–^ mice express green fluorescent protein (GFP) under the control of the respective endogenous serpin promoter, expression of the GFP reporter was quantified in BMDMCs by flow cytometry. In agreement with the transcription data, higher GFP levels were reported in mast cells when driven by endogenous *Serpinb6a* promoter then when driven by the endogenous *Serpinb9a* promoter. Furthermore, *Sb6a*^–/–^ and double-deficient *Sb1a*.*Sb6a*^–/–^ mast cells had similar levels of GFP expression (Figure 2C), indicating that deletion of *Serpinb1a* does not alter basal expression of Serpinb6a in BMDMCs. Co-expression of granule serine proteases and their respective cytosolic inhibitory serpins in BMDMCs suggest cell-intrinsic regulatory mechanisms as shown for other leukocytes (Kaiserman and Bird, 2010; Benarafa and Simon, 2017). We then compared the relative frequency of peritoneal mast cells in mice lacking *Serpinb1a* (*Sb1a*^–/–^), *Serpinb6a* (*Sb6a*^–/–^), *Serpinb9a* (*Sb9a*^–/–^), or both *Serpinb1a* and *Serpinb6a* (*Sb1a*.*Sb6a*^–/–^) and found no statistically significant differences compared to WT mice (Figure 2D and Supplementary Figures 1A,B). Together, these findings show that these cytosolic serpins are not required for development and maturation of mast cells under steady state conditions.

### *Sb9a*^–/–^ Bone Marrow-Derived Mast Cells Are Highly Sensitive to Granule Permeabilization Induced Cell Death

**FIGURE 3:**
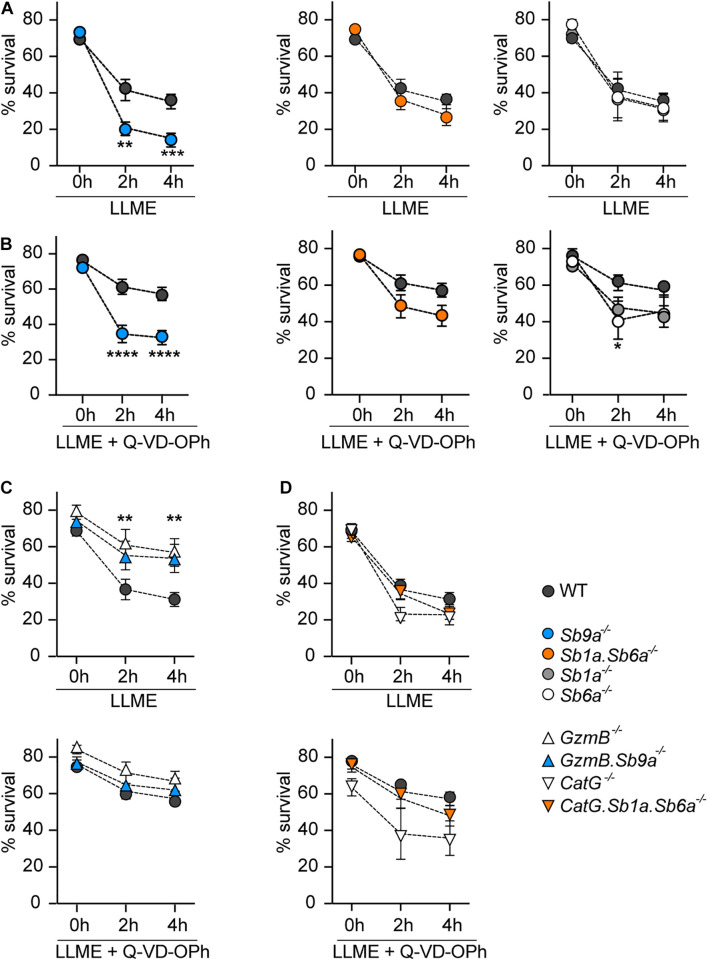
Kinetics of cell death induced by granule permeabilization of BMDMCs. BMDMCs were treated with 250 μM L-leucyl-L-leucine methyl ester (LLME) with or without 50 μM Q-VD-OPh as indicated: **(A)** serpin-deficient BMDMCs, **(B)** serpin-deficient BMDMCs with Q-VD-OPh, **(C)** *GzmB*^–/–^ and *GzmB.Sb9a*^–/–^ BMDMCs with or without Q-VD-OPh, **(D)** *CatG*^–/–^ and *CatG.Sb1a.Sb6a*^–/–^ BMDMCs with or without Q-VD-OPh. **(A–D)** Viability was measured by flow cytometry using annexin V-APC and PI/7-AAD labeling as shown in Supplementary Figure 3. WT BMDMCs in each panel are from paired experiments with the corresponding mutant BMDMCs. Data are shown as mean ± SEM and were analyzed by two-way ANOVA (**p < 0.01; ***p < 0.001; ****p < 0.0001) (*n* = 5–14).

Treatment of WT BMDMCs with LLME rapidly induced cell death (Figure 3A and Supplementary Figure 3). The effect of LLME on WT BMDMCs was partly reduced by caspase inhibition with Q-VD-OPh (Figure 3B). *Sb9a*^–/–^ BMDMCs treated with LLME for 2 and 4 h showed significantly reduced survival compared to WT BMDMCs with and without Q-VD-OPh (Figures 3A,B). In contrast, *Sb1a*^–/–^, *Sb6a*^–/–^, and *Sb1a*.*Sb6a*^–/–^ BMDMCs treated with LLME showed comparable survival to WT BMDMCs in the absence of Q-VD-OPh. Treatment with Q-VD-OPh was less efficient at improving survival in *Sb1a*^–/–^, *Sb6a*^–/–^, and *Sb1a*.*Sb6a*^–/–^ BMDMCs treated with LLME compared to WT, suggesting that multiple proteolytic pathways contribute to the fate of mast cells upon granule permeabilization and that cytosolic serpins, particularly Serpinb9a, are important for survival following leakage of granule proteases into the cytosol.

### Granzyme B Is the Main Inducer of Apoptosis in Mast Cells Upon Granule Leakage

We then investigated BMDMCs of mice deficient for the target proteases of the three serpins. In particular, *GzmB*^–/–^ BMDMCs were highly resistant to LLME-induced cell death compared to WT BMDMCs (Figure 3C). Moreover, *GzmB.Sb9a*^–/–^ BMDMCs had similar resistance to LLME treatment as *GzmB*^–/–^ BMDMCs, and notably demonstrated higher survival than WT BMDMCs. Therefore, the susceptibility of *Sb9a*^–/–^ BMDMCs to granule protease-mediated suicide is principally due to unleashed GzmB activity (Figure 3B). In the presence of Q-VD-OPh, survival improved in WT BMDMCs, reaching the levels of *GzmB*^–/–^ and *GzmB.Sb9a*^–/–^ BMDMCs, suggesting activation of caspases downstream of GzmB (Figure 3C). In contrast, *CatG*^–/–^ BMDMCs had no survival benefit compared to WT BMDMCs (Figure 3D). The survival of *CatG.Sb1a*.*Sb6a*^–/–^ BMDMCs was also similar to WT BMDMCs. Taken together, we conclude that GzmB is the principal mediator of apoptosis following granule leakage in mast cells, and that CatG detectably contributes to cell death only in the absence of its two inhibitory serpins.

### Deletion of Cathepsin G, Granzyme B or Their Inhibitors Does Not Alter Bone Marrow-Derived Mast Cell Differentiation and Apoptosis Induced by Growth Factor Deprivation

**FIGURE 4:**
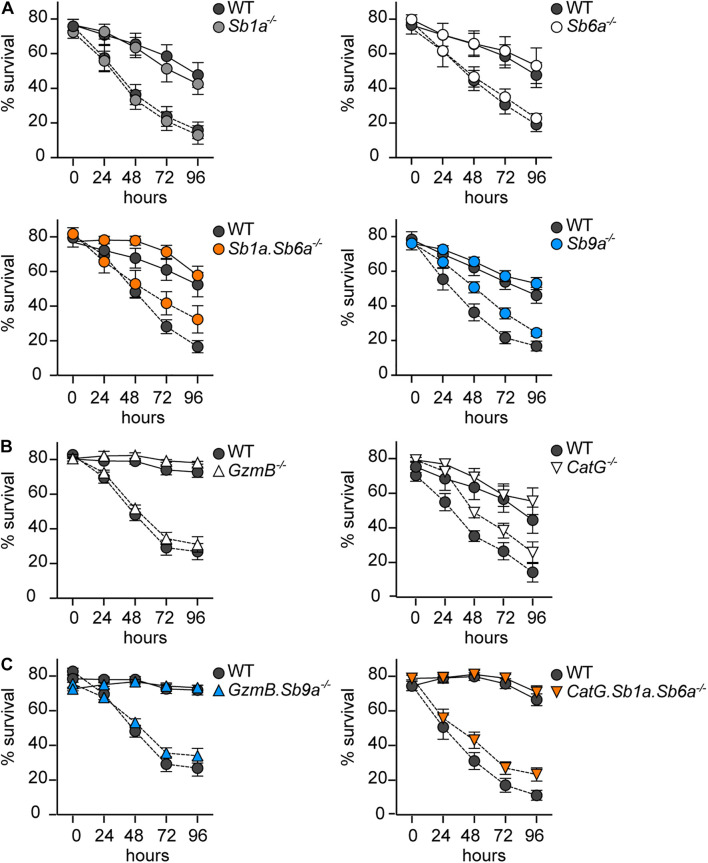
Kinetics of apoptosis of BMDMCs after growth factor deprivation. BMDMCs differentiated *in vitro* for 6–9 weeks were cultured for 96 h in the absence of recombinant IL-3 with (black line) or without (dashed line) of 50 μM Q-VD-OPh. Viability was assessed daily by flow cytometry using annexin V-APC/PI/7-AAD exclusion: **(A)** serpin-deficient BMDMCs, **(B)** *GzmB*^–/–^ and *CatG*^–/–^ BMDMCs, **(C)** *GzmB.Sb9a*^–/–^ and *CatG.Sb1a.Sb6a*^–/–^ BMDMCs. WT BMDMCs in each panel are from paired experiments with the corresponding mutant BMDMCs. Data are shown as mean ± SEM and were analyzed by two-way ANOVA (*n* = 5–15).

Survival and differentiation of BMDMCs *in vitro* is dependent on the supply of growth factors, such as IL-3, which sustain survival in part by inhibiting the intrinsic pathway of apoptosis (Karlberg et al., 2010b; Ottina et al., 2015). To test whether intracellular serpins are also involved in this intrinsic apoptosis pathway, we measured survival of BMDMCs for 96 h after IL-3 withdrawal. Survival of WT BMDMCs was substantially enhanced by the caspase inhibitor Q-VD-OPh (Figure 4). In paired experiments, we observed no significant difference in survival of *Sb1a*^–/–^, *Sb6a*^–/–^, *Sb9a*^–/–^, and *Sb1a*.*Sb6a*^–/–^ BMDMCs compared to WT with or without caspase inhibition (Figure 4A). Similarly, BMDMCs derived from mice lacking GzmB and CatG showed no significant protection against cell death induced by IL-3 withdrawal in presence or absence of Q-VD-OPh (Figure 4B). Finally, we tested the survival of BMDMCs lacking the serpins as well as their respective target protease. *GzmB.Sb9a*^–/–^ and *CatG.Sb1a.Sb6a*^–/–^ BMDMCs were comparable in their survival as paired cultures of WT BMDMCs (Figure 4C). These findings indicate that although IL-3 removal triggers caspase activation and apoptosis, this pathway does not require granule proteases GzmB and CatG, and it is not regulated by cytosolic serpins.

### Granzyme B Triggers the Activation of Caspase-3 Following Granule Leakage

**FIGURE 5:**
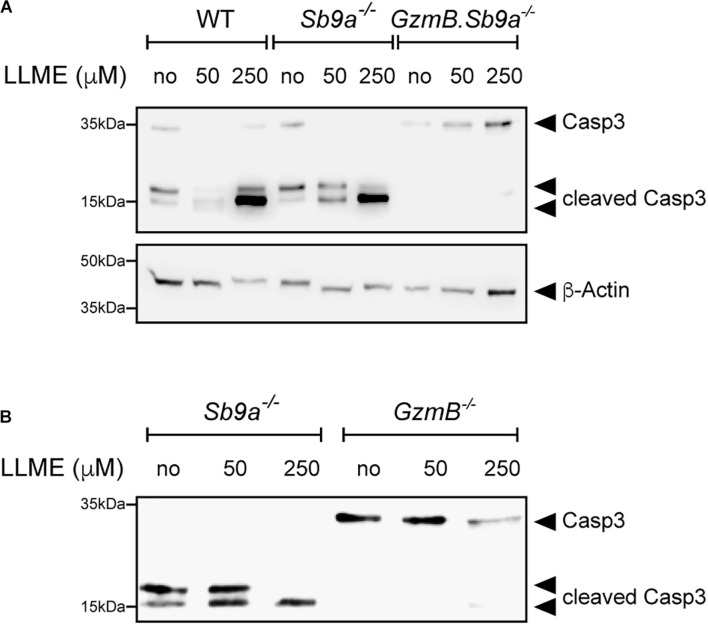
GzmB-dependent cleavage of caspase-3 in LLME-treated BMDMCs. Western Blot analysis of caspase-3 cleavage in BMDMC of **(A)** WT, *Sb9a*^–/–^ and *GzmB.Sb9a*^–/–^ mice **(B)** *Sb9a*^–/–^ and *GzmB*^–/–^ mice stimulated with 50 and 250 μM L-leucyl-L-leucine methyl ester (LLME) for 30 min.

GzmB is a known inducer of apoptosis when delivered with perforin to target cells by cytotoxic T lymphocytes and natural killer cells. In the cytosol, GzmB processes multiple targets including direct proteolytic activation of procaspase-3 and indirect activation via mitochondrial apoptotic events (Lord et al., 2003). As caspase inhibition in *GzmB*^–/–^ BMDMCs treated with LLME only marginally improved survival (Figure 3B), we tested whether GzmB is required for caspase activation upon permeabilization of granules. Western blot analysis of LLME-treated BMDMCs revealed that the active caspase-3 p17 fragment is generated in a dose-dependent manner in WT and *Sb9a*^–/–^ BMDMCs. Most importantly, absence of GzmB in *GzmB*^–/–^ and in *GzmB.Sb9a*^–/–^ BMDMCs prevented the cleavage of pro-caspase-3 p35 into p19 and the active p17 fragment. Conversely, caspase-3 processing was accelerated in *Sb9a*^–/–^ BMDMCs showing a complete conversion into the active p17 fragment even treated at a low LLME concentration (Figures 5A,B). Overall, our findings indicate that GzmB is the most effective serine protease inducing apoptosis in mast cells after leakage from granules. Sb9a acts as a gatekeeper for caspase-3 activation by inhibiting GzmB. The higher survival of *GzmB*^–/–^ compared to WT BMDMCs (Figure 3C) suggest that the protease eventually overwhelms its inhibitor to induce caspase-dependent and caspase-independent cell death.

## Discussion

Mast cells contribute to severe symptoms associated with allergic disorders (Galli et al., 2008). Reducing mast cell numbers at disease-specific locations may therefore provide a novel therapeutic axis (Karra et al., 2009; Hagforsen et al., 2015; Cildir et al., 2017). Studies by Melo et al. (2011a, b) and Paivandy et al. (2014) showed that mast cells are sensitive to death in response to lysosomotropic agents such as LLME and mefloquine (an anti-malaria drug) due to cell-intrinsic leakage of their secretory granules and that this form of cell death is mediated by serine proteases and caspases. However, the serine protease(s) involved in this cell death pathway remain incompletely defined: BMDMCs deficient in the tryptase mMCP-6 were only marginally protected against cell death and deletion of the chymase mMCP-4 or the carboxypeptidase A (CPA) had no effect (Melo et al., 2011b). Here we have confirmed that cell death induced by LMME in human HMC-1.2 and mouse BMDMCs is efficiently blocked by the combined use of inhibitors of caspases and serine proteases.

We found that the serine proteases GzmB and CatG, previously associated with granule-mediated suicide in other leukocyte classes (Kaiserman and Bird, 2010; Benarafa and Simon, 2017), are also present at appreciable levels in mast cells, comparable to levels of the signature mast cell protease, chymase. We hypothesized that these proteases may be involved in LLME-mediated mast cell death, and that their cognate cytosolic serpins might act to block this death pathway. Indeed, *Sb1a* ^–/–^, *Sb6a*^–/–^ and, most severely, double-deficient *Sb1a.Sb6a*^–/–^ neutrophils have increased susceptibility to this form of cell death as both serpins contribute additively to inhibit CatG (Burgener et al., 2016, 2019). Moreover, granule permeabilization induces cell death in LLME-treated neutrophils in a CatG-dependent manner (Baumann et al., 2013; Burgener et al., 2019). Here, we found that Serpinb1a, Serpinb6a (and their target CatG) are highly expressed in mast cells. Yet, Serpinb1a and Serpinb6a had limited protective effects on mast cells following LLME exposure and this protection was only noticeable when caspases were inhibited. *Sb1a.Sb6a*^–/–^ BMDMCs did not show increased death compared to single knockout *Sb1a*^–/–^ or *Sb6a*^–/–^ BMDMCs. CatG deletion rescued this mild phenotype in *CatG.Sb1a.Sb6*^–/–^ BMDMCs, demonstrating that CatG has a real but minor effect on mast cell suicide.

Similarly, Serpinb9 protects activated cytotoxic T lymphocytes and natural killer cells against cell intrinsic death induced by GzmB stored in their granules (Hirst et al., 2003; Ida et al., 2003; Bird et al., 2014; Mangan et al., 2017). GzmB and its inhibitor Serpinb9a are expressed in BMDMCs as well as in human mast cells and cell lines (Bladergroen et al., 2005; Pardo et al., 2007; Strik et al., 2007). Importantly, we found that *GzmB*^–/–^ BMDMCs are resistant to mast cell death induced by LLME and that their survival was significantly improved compared to WT BMDMCs. Conversely, Serpinb9a deletion increased death of BMDMCs. The cytoprotective function of Serpinb9a is principally through inhibition of GzmB since *GzmB.Sb9a*^–/–^ BMDMCs were as resistant to LLME as *GzmB*^–/–^ BMDMCs. We found that Pefabloc (AEBSF) protects against LLME-induced death in WT BMDMCs, yet Pefabloc inhibits most serine proteases but not GzmB. This suggests that other Pefabloc-sensitive mast cell proteases, such as mMCP-6, may contribute in part in this pathway via inactivation of Serpinb9a; such a hypothesis remains to be explored.

Our findings suggest multiple pathways leading to death in mast cells via the activity of granule serine proteases GzmB and, to a lesser extent, CatG. The GzmB pathway appears to be the most prominent as GzmB induces apoptosis even in the presence of its endogenous inhibitor Serpinb9a, whereas CatG appears to be well under control of the abundant basal levels of Serpinb1a and Serpinb6a. The relative contribution of serine proteases thus depends on their abundance, especially relative to the levels of cytosolic serpins and to the expression of downstream targets that will accelerate cellular demise.

Our data indicates that GzmB contributes to the direct or indirect activation of caspase-3 upon granule permeabilization. Yet, caspase inhibition was not sufficient to block cell death. This may be due in part to the ability of GzmB to induce apoptosis via multiple independent pathways (Chowdhury and Lieberman, 2008; Martinvalet, 2015). It has been proposed that pro-caspase-3 is also stored and activated in a granzyme B-dependent manner in mast cell secretory granules (Garcia-Faroldi et al., 2013; Zorn et al., 2013). While we cannot exclude the possibility that granule caspase-3 contributes to mast cell death, our data support the view that caspase-3 activation classically occurs in the cytosol. Indeed, Serpinb9a has a nucleo-cytosolic localization and is not found in granules (Bird et al., 2001), while *Sb9a*^–/–^ BMDMCs treated with LLME showed increased cleavage of caspase-3 into p17 active fragment. The composition of granule cargo is regulated at several levels: the presence of signal peptide is found at the N-terminus of serine and cysteine proteases but not caspases, which are found in the cytosol. The storage of some serine proteases in different granules is further regulated by serglycin. Serglycin is required for the packaging of most mast cell proteases, including chymases and carboxylpeptidase and to a lesser extent tryptases (Ronnberg et al., 2012). GzmB also requires serglycin for localization in granules (Sutton et al., 2016), while CatG does not (Niemann et al., 2007). Interestingly, mast cells of *Serglycin*^–/–^ mice are protected against LLME-induced apoptotic cell death (Melo et al., 2011b). Our findings on GzmB are thus in agreement with previous work demonstrating that a serglycin-dependent protease is responsible for apoptotic death in mast cells following granule permeabilization (Melo et al., 2012).

Mast cells are long-lived in tissues and their survival depends on pro-survival signals and the regulation of the mitochondrial pathway of apoptosis by B-cell lymphoma-2 (Bcl-2) family proteins (Karlberg et al., 2010b; Garrison et al., 2012). Growth factors such as IL-3 and the c-kit ligand stem cell factor (SCF) sustain the survival of mast cells through increased expression of anti-apoptotic Bcl-2 family proteins such as MCL-1 and A1 (Xiang et al., 2001; Reinhart et al., 2018). Conversely, withdrawal of IL-3 or SCF triggers the mitochondrial pathway of apoptosis by increasing the expression of the BH3 only proteins Puma and Bim, respectively (Moller et al., 2005; Ekoff et al., 2007). In support of this model, we found that deletion of Serpinb1a, Serpinb6a and Serpinb9a or their target proteases CatG and GzmB does not alter the cell death kinetics of apoptosis mediated by IL-3 withdrawal. Similarly, *Serglycin*^–/–^ BMDMCs undergo apoptosis normally upon IL-3 withdrawal (Melo et al., 2011b). Targeting mast cells in allergic disorders or in systemic mastocytosis using BH3 mimetics holds promise (Aichberger et al., 2009; Karlberg et al., 2010a; Reinhart et al., 2018). Our findings suggest that induction of GzmB-mediated death in mast cells would provide an additional independent mechanism to target mast cells that could be used in synergy with BH3 mimetics.

In conclusion, we have shown that cytosolic clade B serpins provide a key protective shield in mast cells and prevent the onset of a very rapid apoptotic-like cell death associated with granule serine protease activity. The balance between GzmB and Serpinb9a is particularly important to maintain mast cell survival and the targeted release of GzmB in mast cells and/or downregulation of Serpinb9 may be an interesting therapeutic avenue to target mast cells.

## Materials and Methods

### Mice

All animal studies were approved by the Cantonal Veterinary Office of Bern and conducted in accordance with the Swiss federal legislation on animal welfare. Mice were kept in SPF facilities, in individually ventilated cages, with 12/12 light/dark cycle, autoclaved acidified water, autoclaved cages including food, bedding and environmental enrichment. Age and gender of mice is indicated for each *in vivo* or *ex vivo* model described below as well as in figure legends. All gene targeted mice were on the C57BL/6J background or had been backcrossed to the C57BL/6J background for at least 10 generations. *Sb1a*^–/–^ (*Serpinb1a*^tm1.1Cben^), *Sb6a*^–/–^ (*Serpinb6a*^tm1.1Pib^), *Sb9a*^–/–^ (*Serpinb9a*^tm1.1Pib^) were described previously (Scarff et al., 2003; Benarafa et al., 2007; Rizzitelli et al., 2012). *CatG*^–/–^ (*Ctsg*^tm 1Ley^) and *GzmB*^–/–^ (*Gzmb^tm 1Ley^*) mice were kindly provided by Christine Pham (Washington University, St. Louis, MO) (MacIvor et al., 1999) and Australian Phenome Facility (Heusel et al., 1994), respectively. *Sb1a.Sb6a*^–/–^ mice were generated by mating compound heterozygous F1 mice as described previously (Burgener et al., 2019). All double and triple knockout mice as *GzmB.Sb9a*^–/–^ and *CatG.Sb1a.Sb6a*^–/–^ mice were generated in our facility by compound heterozygote mating.

### Peritoneal Mast Cells and Flow Cytometry

Peritoneal lavage was performed using 5 mL PBS supplemented with 1% heat-inactivated FBS on healthy female and male mice. Total cell numbers were evaluated by counting cells manually in a Neubauer chamber. Relative percentages of live mast cells were determined by flow cytometry; analysis was performed using FlowJo with single cell gating, dead cell exclusion using propidium iodide (PI) and, mast cells identified as FcεRI^+^, IgE^+^, and, c-kit^+^. Single cell suspensions blocked with anti-CD16/CD32 (clone 2.4G2) and stained for 30–40 min on ice with fluorescently labeled antibodies (BioLegend, BD Biosciences) were acquired using a 4-color FACS Calibur (BD Biosciences). Mast cells were permeabilized using the Foxp3 buffer (eBioscience) and stained using an anti-mouse granzyme A antibody (eBioscience, clone 3G8.5).

### Cell Culture

The human mast cell leukemia cell line HMC-1.2 was generated as previously described (Dr. J. Butterfield) and kindly provided by Prof. G. Nilsson (Karolinska Institute, Stockholm) (Butterfield et al., 1988; Nilsson et al., 1994). HMC-1.2 cells were cultured as previously described (Sundstrom et al., 2003). Bone marrow derived mast cells (BMDMCs) were obtained by flushing femurs and tibias bone marrow cells of 6–12 week old female and male mice and cultured in DMEM with 2 mM L-glutamine, supplemented with 10% heat-inactivated FBS, 50 μM β-mercaptoethanol, 1% penicillin/streptomycin, and 10 ng/mL recombinant mouse IL-3 (Peprotech). The cells were maintained at a concentration of 2.5 × 10^5^–1 × 10^6^ cells/mL, at 37°C in 5–7% CO_2_. Maturity was assessed by analysis of the expression of c-Kit, IgE, and FcεRI by flow cytometry 4–5 weeks after isolation (Supplementary Figure 4). In various assays, BMDMCs were seeded at 1 × 10^6^ cells/mL and incubated at indicated concentrations with L-leucyl-L-leucine methyl ester (LLME) (Bachem, # G-2550), Q-VD-OPh (ApexBio, #A1901) or Pefabloc SC (Sigma, #PEFBSC-RO). In some experiments, mature BMDMCs (1.0 × 10^6^/mL) were cultured in absence of rmIL-3 with DMEM, 1% heat-inactivated FBS, 1% penicillin/streptomycin up to 96 h with or without 50 μM Q-VD-OPh (ApexBio, #A1901). Apoptosis and necrosis of mast cells *in vitro* was determined by staining with Annexin V-fluorescein isothiocyanate (FITC) or Annexin V-allophycocyanin (APC) and propidium iodide (PI) or 7-aminoactinomycin D (7-AAD) at RT for 15 min and measured with a 4-color flow cytometer (FACScalibur, BD Biosciences).

### Quantitative RT-PCR

RNA-isolation was performed using RNA-Bee (AMS Biotechnology, #CS-501B). In brief, 3–5 × 10^6^ BMDMCs were resuspended in 1 mL RNA-Bee and total RNA extracted following the manufacturers protocol. After DNAse treatment, 300 ng total RNA was reverse transcribed using ProtoScript III reverse transcriptase and random primer mix (New England Biolabs). Real-time quantitative PCR amplification was carried out in duplicate or triplicate using Mastermix Plus SYBR Assay-Low Rox (Eurogentec, #RT-SY2X-03+WOULRF) in a Vii7 thermocycler (Applied Biosystems). Sequences of the validated specific primers for proteases, serpins and S16 ribosomal protein (RPS16) are shown in Supplementary Table 1 (Chen et al., 2005; Martin et al., 2005; Ekoff et al., 2007; Malbec et al., 2007; Andrew et al., 2008; Cremona et al., 2013). For each sample, mRNA levels were expressed relative to S16 expression.

### Western Blot

BMDMCs were treated for 0.5 h with 50–500 μM L-leucyl-L-leucine methyl ester (LLME) (Bachem, #G-2550) and lysed in 0.1% Triton-X-100 lysis buffer without protease inhibitors followed by 2 × 30 s sonication (Soniprep 150 plus, MSE). Lysates were centrifuged at 10,000 g for 10 min at 4°C and supernatant was collected. Total protein concentration in lysates were determined by the BCA assay (Thermo Fisher Scientific, #23225). Cell lysates were pooled with methanol/chloroform-precipitated cell-free supernatants or cell lysates alone (30 μg total protein) were resolved in SDS-PAGE under reducing conditions using Tris-Glycine Buffer. After transfer on nitrocellulose, blocking was performed using 5% skimmed milk and blots were probed with rabbit anti-mouse caspase-3 (Cell Signaling, #9662) and then stripped and reprobed with anti-β-Actin antibody (Abcam, #ab8227).

### Statistical Analysis

Statistical analysis was performed using Prism 8.0c (GraphPad, San Diego, CA). Non-parametric tests were used to analyze data from *in vivo* and *ex vivo* studies. Independent experiments were performed and pooled, scatter plots represent individual mice and horizontal lines indicate mean ± SEM or median with range. Experiments were analyzed by one-way or two-way ANOVA with Tukey *post-test* and *p*-values of *p* < 0.05 were considered statistically significant (^****^P < 0.0001; ^∗∗∗^*P* < 0.001; ^∗∗^*P* < 0.01; ^∗^*P* < 0.05). Tests used and number of replicates are indicated in figure legends.

## Data Availability Statement

The original contributions presented in the study are included in the article/Supplementary Material, further inquiries can be directed to the corresponding author.

## Ethics Statement

Animal experimentation was conducted in compliance with the Swiss Animal Welfare legislation and animal studies were reviewed and approved by the commission for animal experiments of the canton of Bern, Switzerland under licenses BE8/16 and BE35/19.

## Author Contributions

SB designed, performed the experiments, analyzed the data, and wrote the manuscript. MB, NL, SS, and PB performed the experiments and analyzed the data. TK and PIB provided key reagents, scientific advice, and revised the manuscript. CB supervised the project, designed the experiments, analyzed the data, and wrote the manuscript. All authors contributed to the article and approved the submitted version.

## Conflict of Interest

The authors declare that the research was conducted in the absence of any commercial or financial relationships that could be construed as a potential conflict of interest.

## Publisher’s Note

All claims expressed in this article are solely those of the authors and do not necessarily represent those of their affiliated organizations, or those of the publisher, the editors and the reviewers. Any product that may be evaluated in this article, or claim that may be made by its manufacturer, is not guaranteed or endorsed by the publisher.

## Supplementary Material

Supplementary Figure 1 Analysis of peritoneal mast cells. **(A)** Flow cytometry gating strategy of steady state peritoneal lavage cells to identify mast cells as IgE^+^, FcεR1a^+^, and, c-Kit^+^. **(B)** Mast cells (IgE^+^, FcεR1a^+^, c-Kit^+^) in peritoneal cavity of WT, serpin-deficient and protease-deficient mice. Data were analyzed by one-way ANOVA and no difference was found for any deficient mouse relative to WT mice; data are shown as median (red line) and for individual mice (*n* = 3–11/sex/genotype).

Supplementary Figure 2 BMDMCs and peritoneal mast cells do not express granzyme A protein. **(A)** Representative flow cytometry plots of peritoneal mast cells and identified as IgE^+^, FcεR1a^+^, and, c-Kit^+^. Data are representative of three preparations of C57BL/6 wild-type mice at 6–12 weeks. **(B)** Representative flow cytometry plots of BMDMCs cultured with IL-3 for 5 weeks. **(C)** Representative flow cytometry plots of mouse spleen NK cells and CD8 T cells. Data are representative of four C57BL/6 wild-type (WT) and five *GzmA*^–/–^ mice at 6–12 weeks. **(A–C)** Expression of granzyme A was assessed in fixed and permeabilized cells with a specific antibody.

Supplementary Figure 3 Flow cytometry analysis of mast cells viability. Representative flow cytometry dot plots and gating strategy for evaluating mast cell survival with annexin V and propidium iodide staining. BMDMCs were treated or not with LLME as indicated.

Supplementary Figure 4 Maturity of BMDMCs after 4 weeks of *in vitro* differentiation in presence of IL-3. **(A)** Flow cytometry gating strategy. **(B)** Percentage of IgE^+^FcεR1a^+^c-Kit^+^ BMDMCs of each genotype after 4 weeks of maturation *in vitro* with recombinant mouse IL-3.

Supplementary Figure 5 Uncropped western blot of Figures 1, 5. Representative uncropped western blots.
