# Supplementary figures and images for: Granule Leakage Induces Cell-Intrinsic, Granzyme B-Mediated Apoptosis in Mast Cells
Source: Front Cell Dev Biol. 2021 Nov 8;9:630166. doi: 10.3389/fcell.2021.630166 (PMC8630627; doi:10.3389/fcell.2021.630166)

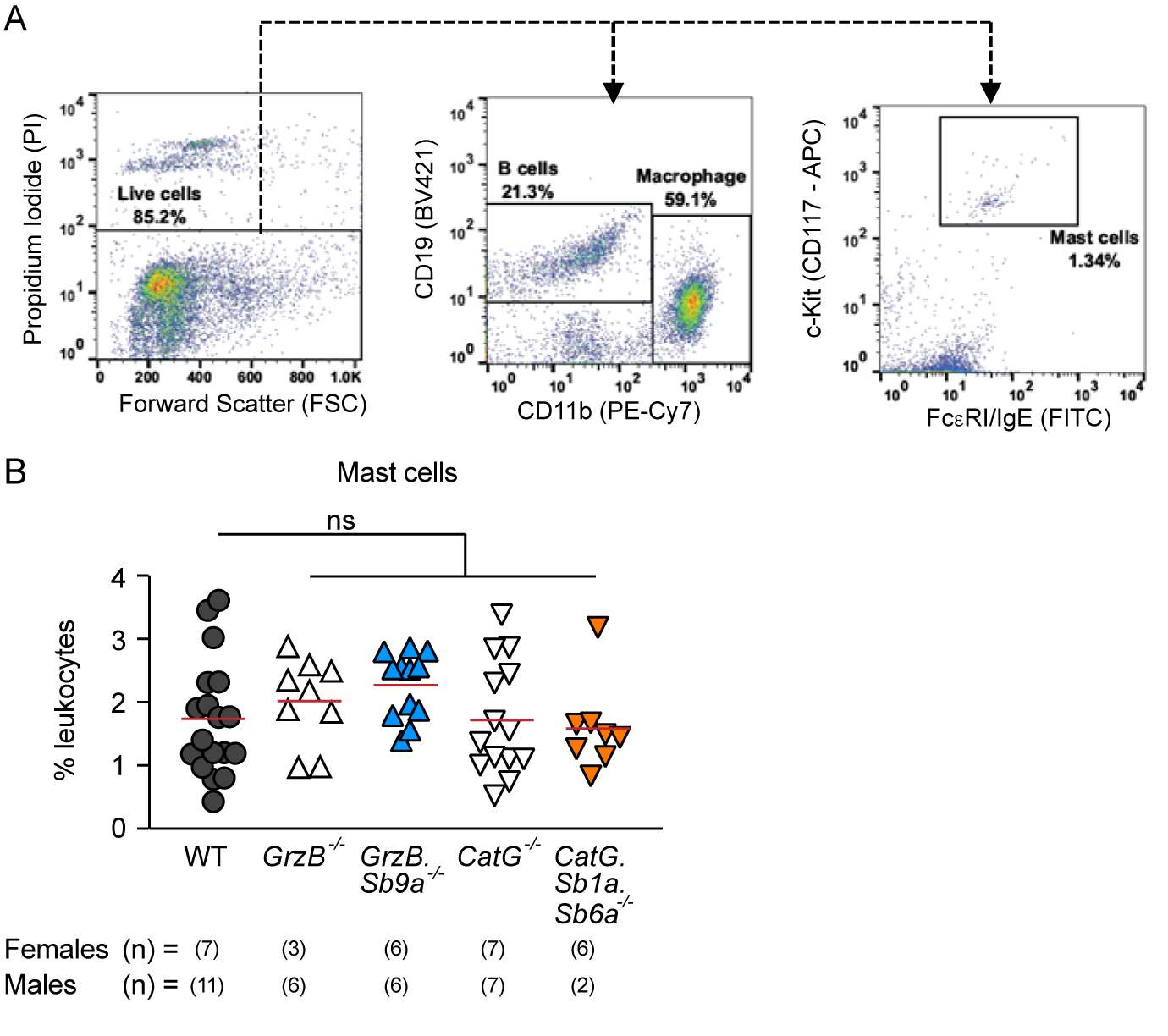

Supplement: Supplementary Figure 1 — Analysis of peritoneal mast cells. (A) Flow cytometry gating strategy of steady state peritoneal lavage cells to identify mast cells as IgE+, FcεR1a+, and, c-Kit+. (B) Mast cells (IgE+, FcεR1a+, c-Kit+) in peritoneal cavity of WT, serpin-deficient and protease-deficient mice. Data were analyzed by one-way ANOVA and no difference was found for any deficient mouse relative to WT mice; data are shown as median (red line) and for individual mice (n = 3–11/sex/genotype). [file Image_1.TIF]

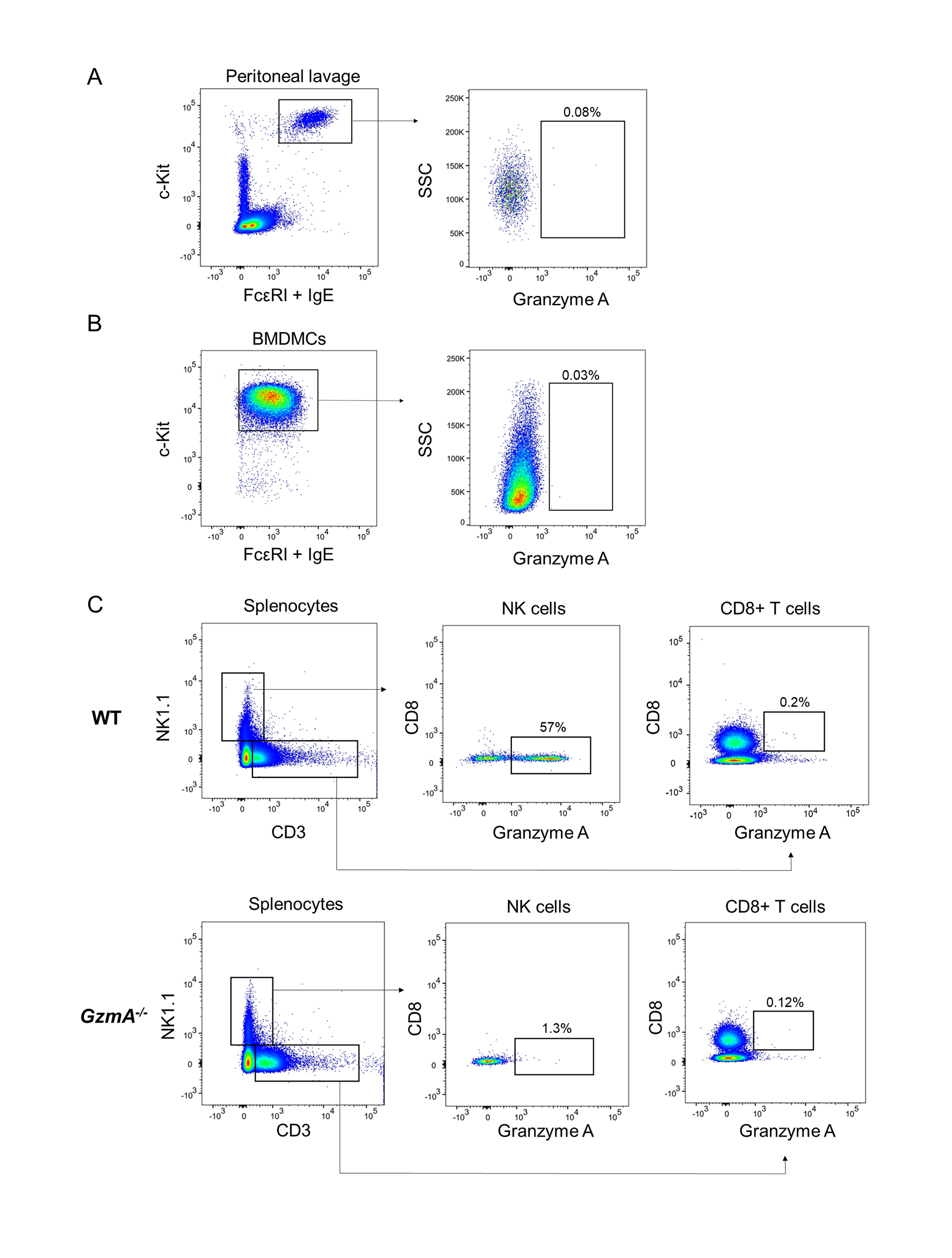

Supplement: Supplementary Figure 2 — BMDMCs and peritoneal mast cells do not express granzyme A protein. (A) Representative flow cytometry plots of peritoneal mast cells and identified as IgE+, FcεR1a+, and, c-Kit+. Data are representative of three preparations of C57BL/6 wild-type mice at 6–12 weeks. (B) Representative flow cytometry plots of BMDMCs cultured with IL-3 for 5 weeks. (C) Representative flow cytometry plots of mouse spleen NK cells and CD8 T cells. Data are representative of four C57BL/6 wild-type (WT) and five GzmA–/– mice at 6–12 weeks. (A–C) Expression of granzyme A was assessed in fixed and permeabilized cells with a specific antibody. [file Image_2.TIF]

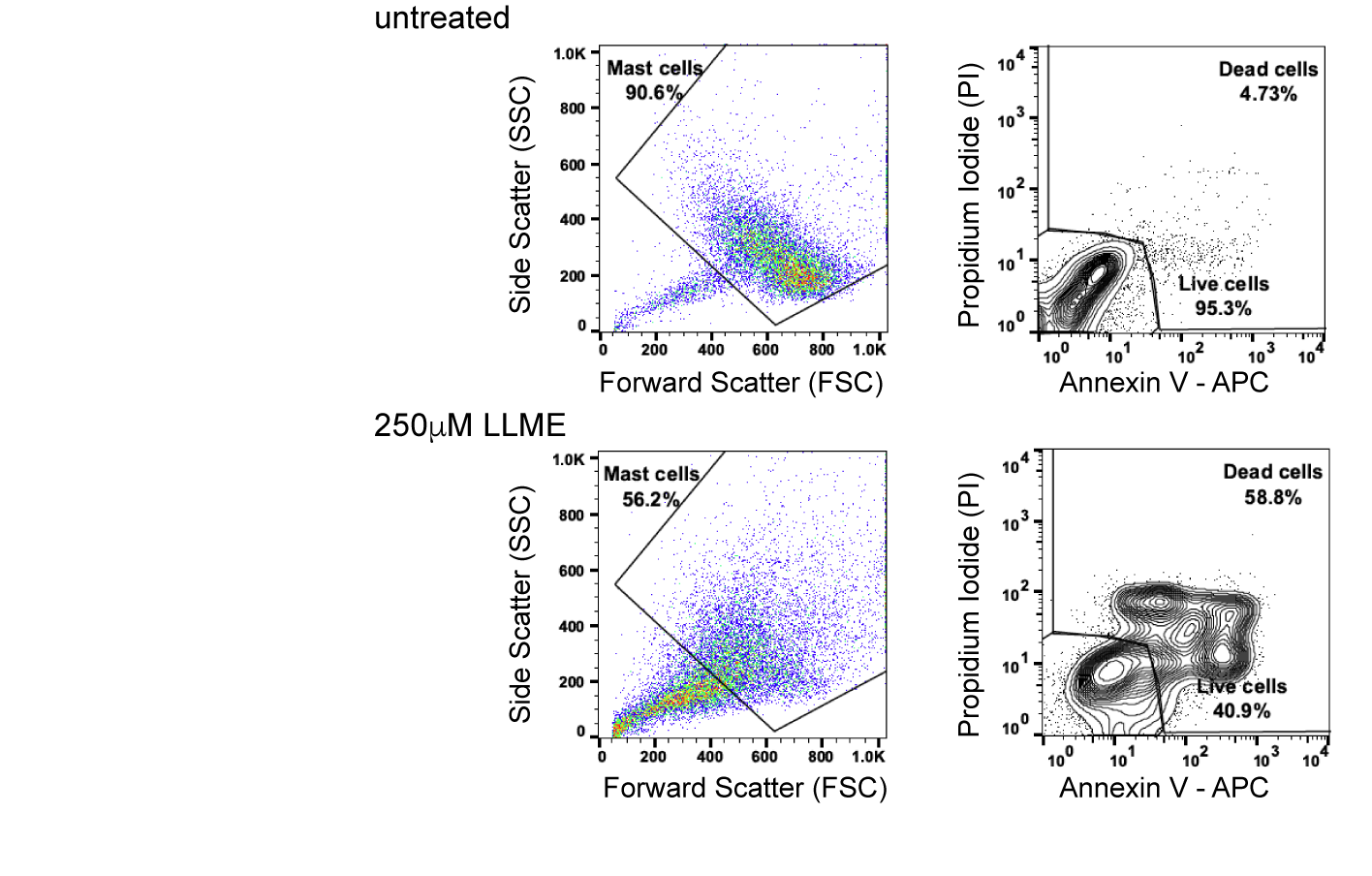

Supplement: Supplementary Figure 3 — Flow cytometry analysis of mast cells viability. Representative flow cytometry dot plots and gating strategy for evaluating mast cell survival with annexin V and propidium iodide staining. BMDMCs were treated or not with LLME as indicated. [file Image_3.TIF]

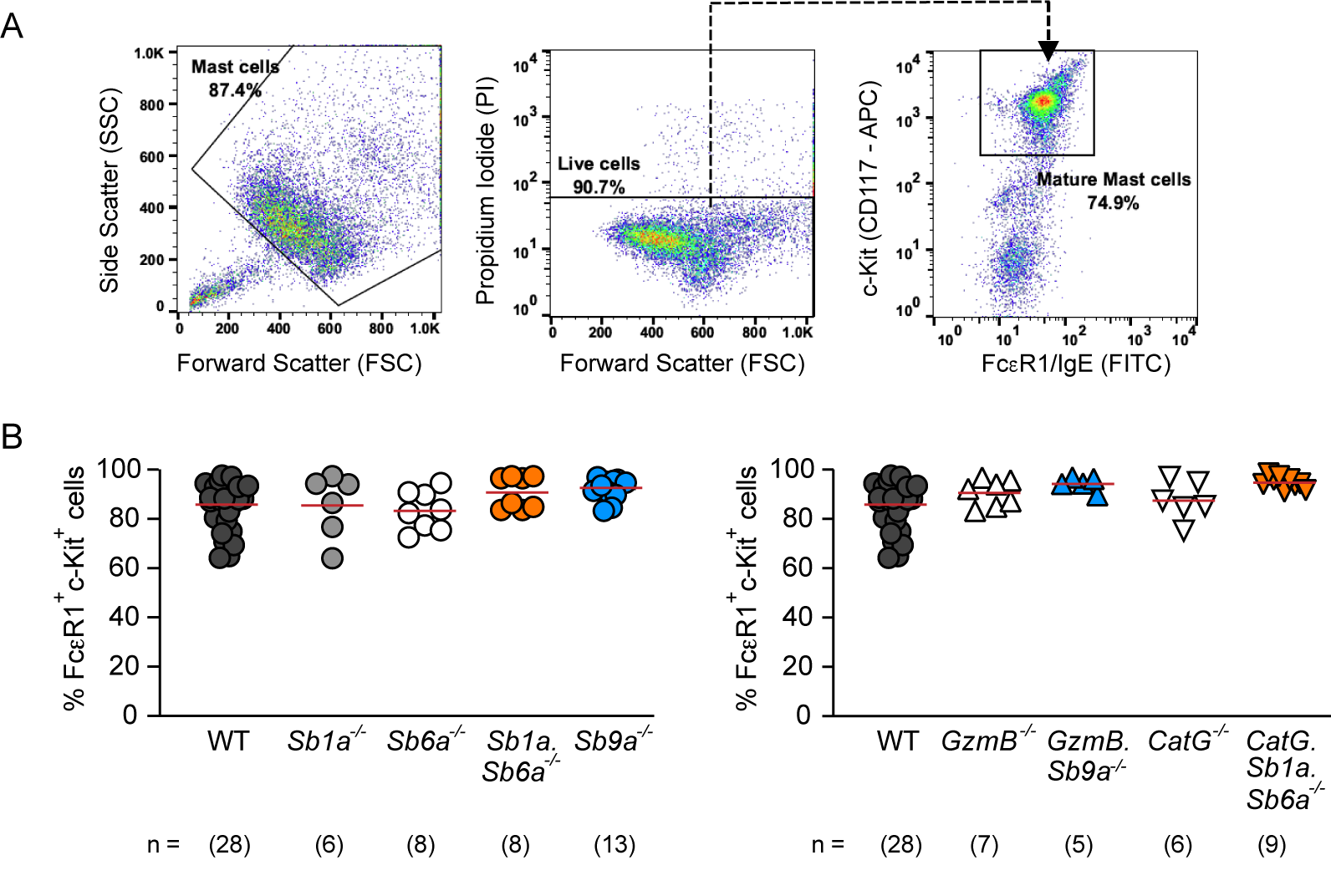

Supplement: Supplementary Figure 4 — Maturity of BMDMCs after 4 weeks of in vitro differentiation in presence of IL-3. (A) Flow cytometry gating strategy. (B) Percentage of IgE+FcεR1a+c-Kit+ BMDMCs of each genotype after 4 weeks of maturation in vitro with recombinant mouse IL-3. [file Image_4.TIF]

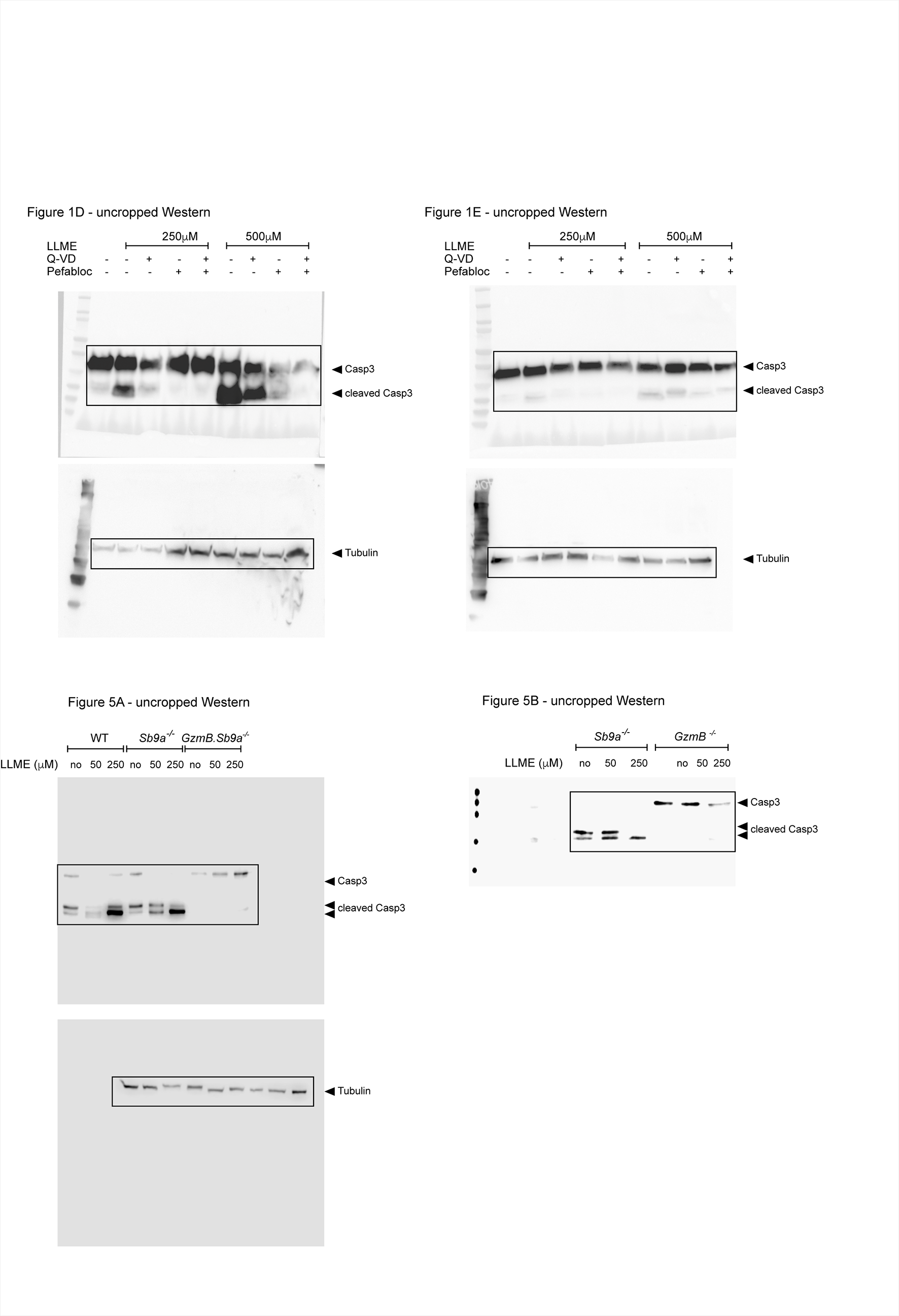

Supplement: Supplementary Figure 5 — Uncropped western blot of Figures 1, 5. Representative uncropped western blots. [file Image_5.TIF]
