# Supplementary material for: Granule Leakage Induces Cell-Intrinsic, Granzyme B-Mediated Apoptosis in Mast Cells
Source: Front Cell Dev Biol. 2021 Nov 8;9:630166. doi: 10.3389/fcell.2021.630166 (PMC8630627; doi:10.3389/fcell.2021.630166)
Supplement: Supplementary file 6 [file Table_1.docx]

**Supplementary Table 1. Primer sequences for RT-qPCR.**

| **Gene** | **Sequence** | ***Ref*.** |
| --- | --- | --- |
| *Rsp16* | F: GAT ATT CGG GTC CGT GTG A  R: TTG AGA TGG ACT GTC GGA TG | (Cremona et al., 2013) |
| *Serpinb1a* | F: CAT CTT CTT CTC TCC CTT CAG C  R: GAG TGT GAG ATG CTC CAC GT | (Cremona et al., 2013) |
| *Serpinb1b* | F: GAG GAG CAA TTC ATG ACA AGA  R: TCT TCT CGC AGA GCA CCT GAA | (Cremona et al., 2013) |
| *Serpinb6a* | F: ATG ACG GTG AGG TGC ATG AG  R: TTA ACA TGG TGA ATG AAG AAA AGG AA | (Cremona et al., 2013) |
| *Serpinb6b* | F: GCA GCC AAT ATA GGT TTT AGG TGT ATG  R: GCT GTG TTG GAT GAA GAA AAG GA | (Cremona et al., 2013) |
| *Serpinb6c* | F: GGC TAC AGC TGC CAC TAC AAT TG  R: GTC TTA ATG TGC TGA ATG AAG AAA ATG | (Cremona et al., 2013) |
| *Serpinb9a* | F: TTC CAC CTT GCT GAG GTC CA  R: CAG TGC AGA TGA TGT GTC GTG | (Andrew et al., 2008) |
| *Ctsg*  (Cathepsin G) | F: AGG GCT GAG TGC TTG TGG  R: AGT TGC TGG GTC CTT TCT CG | This work |
| *Mcpt1*  (mMCP-1) | F: GGA AAA CTG GAG AGA AAG AAC CTA C  R: GAC AGC TGG GGA CAG AAT GGG G | (Ekoff et al., 2007) |
| *Mcpt2*  (mMCP-2) | F: GCC CTA CTA TTC CTG ATG  R: TCT TTC CTG TTT TCC CCC | (Malbec et al., 2007) |
| *Mcpt4*  (mMCP-4) | F: GGT GGT GTT GAG TCT AGA  R: GCA CAT ATG AGG AGA TTC GG | (Malbec et al., 2007) |
| *Cma1*  (mMCP-5) | F: AGG AGC CCA TAA CAA AAC AT  R: TAT TCC AGT TCC AGA TTT CC | (Malbec et al., 2007) |
| *Tpsb2*  (mMCP-6) | F: GCA CTG TCC CTC CTG GCT  R: TGG GAA CCT TCA CTT GCT | (Malbec et al., 2007) |
| *Tpsb1*  (mMCP-7) | F: TCA CTG TGT CCA AAT GCT AA  R: AAG GTG GTT TTC TAT AAT GG | (Malbec et al., 2007) |
| *Mcpt8*  (mMCP-8) | F: CAA CGC TGA AGG AGG GGA  R: TGG GAC ATG CTG CGA CAC | (Malbec et al., 2007) |
| *Mcpt9*  (mMCP-9) | F: CCC ACT GGA ATG AAA AGA  R: TGG CTG TGA GAG AAA AAA | (Malbec et al., 2007) |
| *Mcpt10*  (mMCP-10) | F: GCC CTA CTA TTC CTG ATG  R: GCC TAT CCT TGT AAT GCT | (Malbec et al., 2007) |
| *Cpa3*  (Carboxypeptidase) | F: GCA GGC AGG CAC AGT TAT G  R: TGT TGG TGT TTG GAG AAG AGT C | (Chen et al., 2005) |
| *Gzma*  (Granzyme A) | F: GGG GAT CTA CAA CTT GTA CGG  R: ATT GCA GGA GTC CTT TCC ACC AC | (Martin et al., 2005) |
| *Gzmb*  (Granzyme B) | F: TCA GGC TGC TGA TCC TTG ATC G  R: ATG AAG ATC CTC CTG CTA CTG C | (Martin et al., 2005) |
| *Prf1*  (Perforin) | F: GAG CCC CTG CAC ACA TTA CTG GAA  R: ACA TTC TCA AAG TCC ATC T | (Martin et al., 2005) |

Andrew, K.A., Simkins, H.M., Witzel, S., Perret, R., Hudson, J., Hermans, I.F., Ritchie, D.S., Yang, J., and Ronchese, F. (2008). Dendritic cells treated with lipopolysaccharide up-regulate serine protease inhibitor 6 and remain sensitive to killing by cytotoxic T lymphocytes in vivo. J Immunol *181*, 8356-8362.

Chen, C.C., Grimbaldeston, M.A., Tsai, M., Weissman, I.L., and Galli, S.J. (2005). Identification of mast cell progenitors in adult mice. Proc Natl Acad Sci U S A *102*, 11408-11413.

Cremona, T.P., Tschanz, S.A., von Garnier, C., and Benarafa, C. (2013). SerpinB1 deficiency is not associated with increased susceptibility to pulmonary emphysema in mice. Am J Physiol Lung Cell Mol Physiol *305*, L981-989.

Ekoff, M., Kaufmann, T., Engstrom, M., Motoyama, N., Villunger, A., Jonsson, J.I., Strasser, A., and Nilsson, G. (2007). The BH3-only protein Puma plays an essential role in cytokine deprivation induced apoptosis of mast cells. Blood *110*, 3209-3217.

Malbec, O., Roget, K., Schiffer, C., Iannascoli, B., Dumas, A.R., Arock, M., and Daeron, M. (2007). Peritoneal cell-derived mast cells: an in vitro model of mature serosal-type mouse mast cells. J Immunol *178*, 6465-6475.

Martin, P., Wallich, R., Pardo, J., Mullbacher, A., Munder, M., Modolell, M., and Simon, M.M. (2005). Quiescent and activated mouse granulocytes do not express granzyme A and B or perforin: similarities or differences with human polymorphonuclear leukocytes? Blood *106*, 2871-2878.
